# Access to institutional delivery services and its associated factors among mothers in Jimma Zone, Southwest Ethiopia: a cross-sectional study

**DOI:** 10.1186/s12889-020-09610-8

**Published:** 2020-10-09

**Authors:** Qaro Qanche Kayrite, Waju Beyene Salgedo, Tesfaye Dagne Weldemarium, Shimeles Ololo Sinkie, Dejene Melese Handalo, Teshale Dojamo Obola, Feyera Gebissa Kebene, Muluneh Getachew Garedew, Melaku Haile Likka

**Affiliations:** 1 Department of Public Health, College of Health Science, Mizan-Tepi University, Mizan-Aman, Ethiopia; 2 Department of Health Policy and Management, Faculty of Public Health, Institute of Health, Jimma University, Jimma, Ethiopia; 3 School of Public Health, College of Medicine and Health Sciences, Hawassa University, Hawassa, Ethiopia

**Keywords:** Access, Dimensions, Institutional delivery services, Mothers, Jimma, Southwest Ethiopia

## Abstract

**Background:**

Poor access to institutional delivery services has been known as a significant contributory factor to adverse maternal as well as newborn outcomes. Previous studies measured access in terms of utilization while it has different dimensions (geographic accessibility, availability, affordability, and acceptability) that requires to be measured separately. Therefore, this study was conducted to assess the four dimensions of access and factors associated with each of these dimensions.

**Methods:**

*C*ommunity-based cross-sectional study design was used, employing both quantitative and qualitative methods. A simple random sampling technique was used to select 605 mothers who had given birth in the last 6 months preceding the study. Multi-variable binary logistic regression was used to select factors associated with the four dimensions of access by using AOR with 95% CI. Ethical approval was secured from Jimma University Institutional Review Board.

**Results:**

Five hundred and ninety-three mothers involved in this study, resulting in a response rate of 98%. Four hundred five (68%), 273(46%), 279(47%), and 273(46%) had geographic, perceived availability, affordability, and acceptability access to institutional delivery services, respectively. Antenatal care [AOR = 3.74(1.56, 8.98)], occupation of mother [AOR = 5.10(1.63, 15.88)], and residence [AOR = 1.93(1.13, 3.29)] were independently associated with geographic accessibility. Household graduation [AOR = 1.46(1.03, 2.06)], residence [AOR = 1.74(1.17, 2.59)], and ANC [AOR = 3.80(1.38, 10.50)] were independently associated with perceived availability. Moreover, wealth quintile [AOR = 11.60(6.02, 22.35)], ANC [AOR = 3.48(1.36, 9.61)], and occupation of husband [AOR = 3.63(1.51, 8.74)] were independently associated with affordability. Lastly, mother’s education [AOR = 2.69(1.42, 5.09)], residence [AOR = 2.60(1.66, 4.08)], and household graduation [AOR = 3.12(2.16, 4.50)] were independently associated with acceptability of institutional delivery services.

**Conclusions:**

Moderate proportions of mothers have geographic accessibility to institutional delivery services, but access to the other three dimensions was low. ANC visits of 4 or above, occupation of husband, urban residence, graduation of mother’s household as a model family, higher wealth quintiles, and maternal educational level significantly affect access to institutional delivery services. Thus, it was recommended that concerned bodies should give due attention to ANC services, female education, training of model families, and enhancement of household wealth through job creation opportunities to increase access to institutional delivery services.

## Background

Access to health care represents the ability of people to use health care and shows people’s capacity to benefit from services provided by the healthcare, in light of people’s circumstances and experiences with regard to the health care system. Policies dealing with access to healthcare put greater responsibility on decision-makers to not only make services available but also to actively enable individuals to utilize those services when the need arise. Differences in access (i.e., empowerment) between individuals cause differences in the use of healthcare services [1].

Access to health care has four dimensions: geographic (or physical) accessibility, availability, affordability (or financial access), and acceptability (or cultural access). Geographic accessibility is concerned with the relationship between the location of healthcare facilities (supply factor) and the location of those who need these services and their transportation opportunities (demand factors). Availability includes issues such as the level of supply of staff or drugs, the degree of fit between the hours of service of health care facilities (opening hours) and the times that individuals need services to be provided. Affordability is concerned with the degree of fit between the full costs to the individual using the service and the individual’s ability to pay in the context of the household budget and other demands on that budget. Acceptability is concerned with the fit between provider and patient attitudes toward and expectations of each other [1, 2].

Generally, access can be looked at from two perspectives, each having four dimensions in them. These are the demand-side and supply-side perspectives. Factors in the demand-side influence health service users at all levels, and include physical accessibility, perceived availability, affordability, and cultural acceptability of services. Supply-side factors function at the service delivery level and are not under the control of users. As for demand-side perspective, supply-side factors also include the four access dimensions [3, 4].

Factors that affect access to institutional delivery services (IDS) can be categorized under the demand and supply-side perspectives, and some of these include service location/distance, means of transportation, education, income, information, decision-making power, waiting time, users attitudes and expectations, and provider attitudes among others [3–6].

Ideally, healthcare services must be physically accessible and available, economically affordable, and psychosocially acceptable to communities and societies all the times. This is extremely important for vulnerable populations with specific needs, such as pregnant and delivering mothers [4].

Poor access to IDS has been known as a significant contributory factor to adverse maternal as well as newborn outcomes in Sub-Saharan African countries, including Ethiopia [5]. Worldwide, maternal deaths related to pregnancy and child birth is unacceptably high. In the year 2010, pregnancy and childbirth has contributed for the death of 287,000 women. Developing countries account for almost all maternal deaths (99%), and sub-Saharan Africa takes more than half of that share [7].

In Ethiopia, poor access to IDS is reflected by its high maternal mortality ratio (MMR). According to the 2016 Ethiopian Demographic and Health Survey (EDHS), MMR was estimated to be 412 per 100,000 live births [8].

Health facility delivery is one of the golden intervention strategies to minimize maternal death. It ensures safe delivery, reduces complications, minimizes maternal death, and increases the survival of newborns [9]. Despite this fact, home delivery is not unfamiliar in many developing countries, including Ethiopia. Although institutional delivery has been promoted in Ethiopia, home delivery remains common, primarily in hard-to-reach areas. The 2016 EDHS showed that only 26% of live births were delivered in a health facility in the 5 years before the survey. For rural women, the report showed that only 20% of the births took place in a health facility and 80% of them delivered at home. It was evidenced from the report that institutional delivery varies across different Regional States of the country. For Oromia Regional State, institutional delivery was only 18.8%, which is the third-lowest slightly above Afar (14.7%) and Somali (17.9%) Regional States [8].

Studies conducted in different parts of the Oromia Regional State reported that institutional delivery ranges from 12.3% in Munisa Woreda of Arsi Zone to 47% in Goba Woreda of Bale Zone [6, 10–13]. These studies show that significant proportions of mothers in the Oromia Regional State yet have no access to IDS, and more than half of the births are taking place outside the health facilities without the help of health professionals where the risk of maternal death is very high.

The Ethiopian government has developed an ambitious plan, the Health Sector Transformation Plan (HSTP), which aims to reduce the maternal mortality ratio from 420 per 100,000 live births to 199 per 100,000 live births and to increase deliveries attended by skilled health personnel to 90% by the end of 2020 [14]. However, these targets will never be met unless the level of current access to IDS is known and the factors that affect access are clearly identified in order to design and implement appropriate intervention measures.

There has been substantial research and literature on institutional delivery service utilization and barriers to institutional delivery service utilization, as it is an important international public health issue. However, it is not appropriate to measure access in terms of utilization as access has different dimensions that need to be measured separately. Moreover, there were no previous studies that have specifically focused on the four dimensions of access.

Therefore, the main aim of this study was to determine the four dimensions of access to IDS and identify factors associated with each of these dimensions.

Hence, the result of this study is expected to contribute to fill the gap in the lack of evidence on the four dimensions of access to institutional delivery services. Moreover, the result will also consolidate existing knowledge by examining barriers to access to IDS from a more comprehensive viewpoint.

Lastly, but not least, findings from this study could potentially help policy makers, local decision-makers, and program planners to design specific intervention strategies that will improve access to institutional delivery services, and ultimately improve maternal and newborn health conditions.

## Methods

This study was conducted in six randomly selected Woredas of Jimma Zone (Mencho, Gomma, Nano Benja, Seka Chokorsa, Gumay, and Kersa), Southwest Ethiopia, from March 16 to April 15, 2018. Jimma Zone is one of the 17 Zones found in Oromia Regional State, located 350 km Southwest of Addis Ababa, the capital city of Ethiopia. There are 20 Woredas and two town administrations with a total of 561 Kebeles (the smallest geographic administrative unit in Ethiopia) in Jimma Zone. According to Jimma Zone Health Department annual estimate for the year 2017, the total population of the Zone was 3,312,914, of which 603,222 were reproductive age women. Concerning health facilities, there was 1 tertiary hospital, 8 primary hospitals, 122 health centers, and 513 health posts in the Zone.

A community-based cross-sectional study design was used, employing both quantitative and qualitative methods, to undertake this study. The qualitative method was used to support the findings of quantitative results in order to explore and understand what is behind mere quantitative figures.

The source population for this study was all women of reproductive age group (15–49 years), and the study population was randomly selected mothers who lived for at least 6 months in the selected Kebeles (6 months stay is considered as permanent residence in Ethiopia), and who had given birth in the last 6 months preceding the study (6 months time period preceding the study was preferred to minimize recall bias).

A single population proportion formula was used to determine sample size with assumptions of *P* = 50% (proportion of mothers having access to IDS), 95% confidence level, and 5% margin of error. A design effect of 1.5 was used to account for the sampling variability of multi-stage sampling. The calculated sample size was 576 and 5% added for an expected non-response rate to have a final sample size of **605** for the quantitative study**.**

For qualitative data, four focused group discussions (FGDs) were carried out: two with women health development army leaders, one with home-delivered mothers and one with health facility-delivered mothers. The FGD participants were identified by the suggestion of Health Extension Workers (HEWs) and community elders. Each FGD consisted of 8–10 mothers.

A multi-stage sampling technique was employed, taking the Woredas as primary sampling units (PSU), Kebeles from the selected Woredas as secondary sampling units (SSU), and mothers at households from the selected Kebeles as tertiary sampling units (TSU). Thirty percent of the Woredas and Kebeles were selected by lottery method. Then, depending on the number of mothers who had given birth in the last 6 months preceding the study, the sample size was proportionally allocated to the selected Kebeles. Finally, a simple random sampling (SRS) method was used to get mothers for an interview at a household (HH) level.

Data collection tools were prepared in English and then translated to the local language, i.e. Afan Oromo, by a language expert, and then back to English by another language expert to ensure consistency. Training was given to data collectors and supervisors before data collection about the aim of the study and data collection procedures.

Six data collectors who were fluent in speaking and writing the local language participated in the data collection process using a pretested interviewer-administered structured questionnaire for the quantitative part. For the qualitative part, an interview guide was used for the FGD, and the discussions were recorded using a tape recorder. In addition, hand-written notes were taken. The FGDs were moderated by supervisors who were fluent in speaking and writing the local language.

To ensure data quality, a pretest was conducted in a Woreda which was not included in the study, on 10% of the sample size to determine the clarity of the items and the consistency of the responses. During actual data collection, the filled questionnaires were checked for consistency and completeness each day and submitted to the supervisors. Data were entered into epi-Data version 3.1 by two persons separately and the two files were validated for consistency, and necessary corrections were made before exporting the data into SPSS for analysis.

The dependent variables of measurement were geographic accessibility, perceived availability, affordability, and acceptability, whereas the independent variables were socio-demographic variables (age, religion, ethnicity, residence,marital status), socio-economic variables (HH wealth index, mother’s education, husband’s education, mother’s occupation, husband’s occupation), obstetric and related variables (parity, ANC use), and household-level variables (HH headship, family size, HH model status).

### Operational definitions

1. **Access to institutional delivery service**: - In this study, access to institutional delivery service was measured by its four dimensions separately: geographic accessibility, availability, affordability, and acceptability.
2. **Institutional delivery**: - Is the delivery of a baby that takes place in a health facility, both public and private facilities.
3. **Geographic accessibility**: - Is travel time from mothers’ homes to the nearest health facility on foot walk. In this study, mothers who lived within a distance of 60 min on foot walk from the nearest health facility were considered to have geographic accessibility [15–17]; otherwise not accessible.
4. **Perceived availability**: - In this study, the availability of institutional delivery services was measured from the mothers’ perspective using ten items that assessed mothers’ perceived availability of institutional delivery services, with some major components being availability of: qualified professionals, drugs and supplies, and laboratory services. The items were measured on a 5 points agreement Likert scale, and mean scores were calculated for each mother. Finally, the mean score for the total sample was computed and if a mother’s mean score was greater than or equal to the sample mean, then she was considered to have perceived availability; otherwise not.
5. **Affordability**: - This is when mothers or families are able and willing to pay for the cost of transportation, prescribed medication/services, staying cost at a facility, and cover the opportunity costs related to health facility delivery without any borrowing or selling of basic household resources. There were four items that assessed these four types of costs. These items included whether mothers have the ability and willingness to afford the direct cost of prescribed medicines and supplies, indirect costs related to transportation and staying at the facility, and the presence of family members to accompany them to the facility. These costs should be afforded without borrowing money or selling basic household assets. If the mother was able and willing to pay for all the four types of cost, she was considered able to afford; otherwise not.
6. **Acceptability**: - This is when the available delivery services meet the social and cultural expectations of mothers, families, and communities; and the major components include decision-making power of mothers on the place of delivery, perceived/experienced cleanliness of health facilities, fear of interference by strangers, fear of maltreatment by health professionals, discrimination, and stigma. Fifteen items were developed and measured on 5 points agreement Likert scale, and mean scores were calculated for each mother. Finally, the mean score for the total sample was computed and if a mother’s mean score was greater than or equal to the sample mean, then she was considered as have acceptability of institutional delivery services; otherwise not.
7. **Household wealth index**: - This is the households’ living status; the index was constructed by principal component analysis (PCA) using data on housing conditions, ownership of assets, presence of functional latrine, source of drinking water, ownership of domestic animals, ownership of farmland, and amount of grain harvested in the last production year, among others.

With regard to data processing and analysis, the collected data were checked for consistency, then coded and entered into epi-Data version 3.1. After validation of duplicated files for consistency, it was exported to SPSS version 20 for cleaning and analysis.

When using PCA, all assumptions of PCA such as metric level or dummy coded variables, minimum sample size of 50, cases to items ratio of 5 to 1 or more, two or more correlations of 0.3 or more on the correlation matrix, removing items with sampling adequacy less than 0.5, and significance of Bartlett test of sphericity were checked. Varimax rotation was used to get better loading variables for the components. Additionally, variables with communality less than 0.5 were removed, and then variables with complex structures (having loadings of 0.4 or more on more than one component) on the rotated component matrix were removed. Components with only one variable loading on them were also checked and removed. Finally, seven components were formed, and explained 67% of the total variability. The reliability of variables within each component was assessed, and all of the variables in the components had Cronbach alpha of 0.6 and above.

For the descriptive statistical part, means, proportions, tables, graphs, and charts were used to summarize and present the results of this study. To identify associated factors, first, the chi-square test was run for individual study variables for each of the access dimensions. Variables that fulfilled the chi-square assumptions were passed for simple logistic regression. Then, variables with a *p*-value ≤0.25 on simple logistic regression were taken as candidates for multivariable logistic regression using 10% for probability of entry and 15% for probability of removal. A backward likelihood ratio method was used for variable selection. Finally, four separate models were fitted to show the effect of significant predictor variables on the outcome variables at a p-value ≤0.05. The adequacies of the models were checked by the Hosmer and Lemeshow test of goodness of fit at p-value ≥0.1. Nagelkerke pseudo R-squared and the overall prediction powers of the models from the classification tables were also reported. Using an adjusted odds ratio (AOR) with 95% confidence interval, the associations of dependent and independent variables were interpreted.

Qualitative data were first transcribed, coded, and categorized to form primary themes based on the objectives of the study. Besides, quotes of participants that supported or contradicted the key quantitative findings were triangulated with the quantitative findings during the discussion of the results.

## Results

### Socio-demographic and economic characteristics of mothers

Out of the 605 total sample size, 593 mothers participated in this study, resulting in a response rate of 98%. The mean age of the mothers was 31 years (±6 SD), and 36% of them were in the age group 26–30 years. Majority of the mothers were Muslim in religion and more than three-fourths of them were Oromo in ethnicity. About 77% of the mothers lived in rural areas, and nearly all of the mothers (98%) were currently in marriage. Approximately half of them were housewives, followed by farmers. Thirty seven percent of the mothers can read and write, whereas only 20% of them attended secondary education and above. Husbands were slightly more educated then mothers (Table 1).

**Table 1:** Socio-demographic and economic characteristics of mothers in Jimma Zone, Southwest Ethiopia, 2018 (*n* = 593)

Characteristics	Category	Frequency(%)
Age of mother	≤20	27 (4.6)
	21–25	99 (16.7)
	26–30	211 (35.6)
	31–35	147 (24.8)
	≥36	109 (18.4)
Religion	Muslim	436 (73.5)
	Orthodox	104 (17.5)
	Others^a^	53 (9)
Ethnicity	Oromo	460 (77.6)
	Dawuro/Wolaita	47 (8)
	Amhara	43 (7)
	Yem/Keffa	25 (4)
	Others^b^	20 (3.4)
Residence	Rural	454 (76.6)
	Urban	139 (23.4)
Marital status	Married/cohabiting	583 (98.3)
	Divorced/widowed	10 (1.7)
Type of marrige	Monogamous	541 (91.9)
	Polygamous	47 (8.1)
Mother’s occupation	Housewife	292 (49.2)
	Farmer	229 (38.6)
	Merchant/laborer	40 (6.8)
	Employee	32 (5.4)
Husband’s occupation	Farmer	413 (69.6)
	Merchant	78 (13.2)
	Laborer	51 (8.6)
	Employee	44 (7.4)
	Others^c^	7 (1.2)
Mother’s educational status	Can’t read &write	90 (15.2)
	Can read & write	222 (37.4)
	Primary education	164 (27.7)
	Secondary or above	117 (19.7)
Husband’s educational status	Can’t read &write	43 (7.3)
	Can read & write	194 (32.7)
	Primary education	223 (37.6)
	Secondary or above	133 (22.4)
Household wealth index	Lowest quintile	118 (19.9)
	Second quintile	119 (20.1)
	Middle quintile	119 (20.1)
	Fourth quintile	119 (20.1)

^a^Protestant & Waqafata, ^b^Tigre, Gurage & Silte, ^c^Student, Sheik & Priest

### Household-level characteristics of mothers

The average family size was 5.46 persons per household. More than half of the mothers’ households had 5 or fewer members in the family, and the majority of the households were headed by husbands. Forty seven percent of the mothers’ households had completed training on the 16 packages of the Ethiopian health extension program and have graduated as model families (Table 2).

**Table 2:** Household level characteristics of mothers in Jimma Zone, Southwest Ethiopia, 2018 (n = 593)

Characteristics	Category	Frequency(%)
Family size	≤5	330 (55.6)
	> 5	263 (44.4)
Head of household	Husband	571 (96.3)
	Self	22 (3.7)
Graduation of mother’s HH as a model family	Yes	280 (47.2)
	No	313 (52.8)

### Obstetric and related characteristics of mothers

**Fig. 1:**
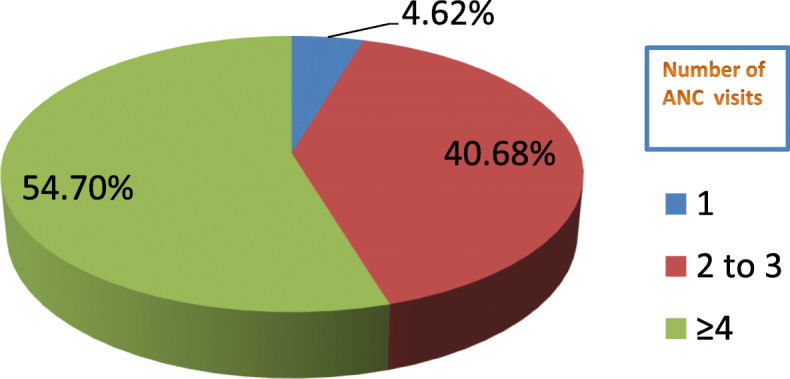
Number of ANC visits during last pregnancy among mothers in Jimma Zone, Southwest Ethiopia, 2018

The average number of children per mother was 3.4, and 395(66.6%) of the mothers had 2–4 children, 135(22.8%) had 5 or more children, and 63(10.6%) of the mothers had one child. Almost all of the mothers, 585(99%) had a history of ANC follow-up for their last pregnancy. Among those who had ANC follow-up, 320(54.7%) had 4 or more visits, 238(40.7%) had 2–3 visits, and 27(4.6%) had only a single visit during their last pregnancy (Fig. 1).

### Dimensions of access to institutional delivery services

**Fig. 2:**
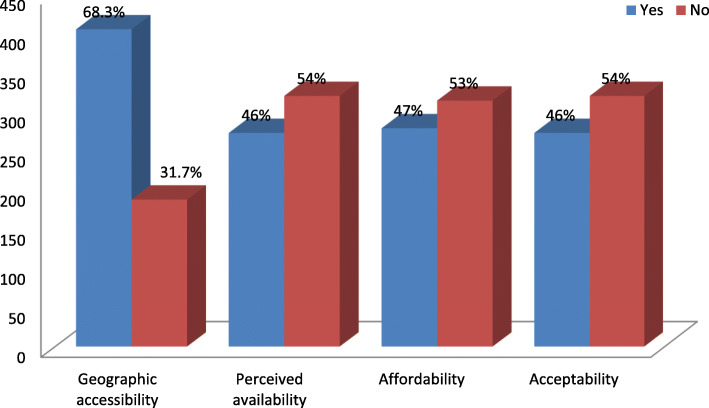
Dimensions of access to institutional delivery services among mothers in Jimma Zone, Southwest Ethiopia, 2018

Slightly more than two-thirds of the total respondents had geographic access to IDS, whereas in the other three dimensions of access, less than half of the mothers had access to IDS. Fourty six percent, 47%, and 46% of the mothers had perceived availability, affordability, and acceptability of IDS, respectively (Fig. 2).

### Factors associated with geographic accessibility to IDS

From the simple logistic regression analysis, the following 12 variables: religion, ethnicity, residence, mother’s occupation, husband’s occupation, mother’s educational level, husband’s educational level, family size, household model status, parity, number of ANC visits, and wealth index were found to be candidates for multivariable logistic regression. After running multivariable logistic regression, mothers’ occupation, residence, and ANC visits during the last pregnancy were found to be independently associated with geographic accessibility to IDS (*p* ≤ 0.05, Table 3).

**Table 3:** Multivariable logistic regression of factors associated with geographic accessibility to institutional delivery services among mothers in Jimma Zone, Southwest Ethiopia, 2018

Variables	Travel time to the nearest health facility on foot walk		COR (95% CI)	AOR (95% CI)
	> 60 mins	≤ 60 mins		
Occupation of mother				
Farmer**	100 (16.9%)	129 (21.8%)	1.0	1.0
Housewife	75 (12.6%)	217 (36.6%)	2.24 [1.54, 3.24]*	2.33 [0.54, 3.54]
Merchant	1 (0.2)	20 (3.4%)	15.50 [0.04, 22.74]	15.47 [0.97, 20.99]
Laborer	8 (1.3%)	11 (1.9%)	1.06 [0.41, 2.74]	1.14 [0.41, 3.17]
Employee	4 (0.7%)	28 (4.7%)	5.42 [1.84, 15.97]*	5.10 [1.63, 15.88]*
Residence				
Rural **	160 (27.0%)	294 (49.6%)	1.0	1.0
Urban	28 (4.7%)	111 (18.7%)	2.15 [1.36, 3.40]*	1.93 [1.13, 3.29]*
Number of ANC visits				
1**	15 (2.6%)	12 (2.0%)	1.0	1.0
2–3	72 (12.3%)	166 (28.3%)	2.88 [1.28, 6.46]*	3.0 [1.26, 7.17]*
4 or above	99 (16.9%)	222 (37.9%)	2.79 [1.26, 6.77]*	3.74 [1.56, 8.98]*

*Statistically significant at p < 0.05 **Reference category Classification power = 73% Hosmer and Lemeshow test: p = 0.624 Nagelkerke R square = 0.78

This study found that mothers’ occupation was significantly associated with geographic accessibility to institutional delivery services. Employed mothers (government employed or in a private sector other than own business; and referred to as “employed” here after) were 5 times more likely to have geographic accessibility to institutional delivery services when compared to mothers who were farmers (AOR = 5.10[1.63, 15.88]). Another variable that was associated with geographic accessibility was mothers’ residence. Mothers who live in urban areas were about 2 times more likely to have geographic accessibility to institutional delivery services when compared to mothers living in rural areas (AOR = 1.93[1.13, 3.29]).

The number of ANC visits during the last pregnancy was also another variable that was significantly associated with geographic accessibility to institutional delivery services. Mothers who had 4 or more ANC visits during their last pregnancy were about 4 times more likely to have geographic accessibility to institutional delivery services when compared to mothers who had only a single ANC visit during their last pregnancy (AOR = 3.74[1.56, 8.98]). Moreover, mothers who had 2–3 ANC visits during their last pregnancy were 3 times more likely to have geographic accessibility to institutional delivery services when compared to mothers who had only a single ANC visit during their last pregnancy (AOR = 3.0[1.26, 7.17]).

### Factors associated with perceived availability of institutional delivery services

Ten variables were identified as candidates for multivariable logistic regression, and after running multivariable logistic regression, three variables were found to be significantly associated with the perceived availability of IDS. These variables are the number of ANC visits, residence, and model status of a mother’s household (Table 4).

**Table 4:** Multivariable logistic regression of factors associated with perceived availability of institutional delivery services in Jimma Zone, Southwest Ethiopia, 2018

Variables	Perceived availability		COR (95% CI)	AOR (95% CI)
	No	Yes		
Number of ANC visits				
1**	22 (3.8%)	5 (0.9%)	1.0	1.0
2–3	143 (24.4%)	95 (16.2%)	2.92 [1.07, 7.98]*	2.59 [0.94, 7.14]
≥ 4	150 (25.6%)	171 (29.1%)	4.98 [1.84, 13.49]*	3.80 [1.38, 10.50]*
Residence				
Rural **	261 (44.0%)	193 (32.5%)	1.0	1.0
Urban	59 (9.9%)	80 (13.5%)	1.83 [1.24, 2.69]*	1.74 [1.17, 2.59]*
Graduation of mother’s HH as a model family				
No **	187 (31.5%)	126 (21.2%)	1.0	1.0
Yes	133 (22.4%)	147 (24.8%)	1.64 [1.18, 2.27]*	1.46 [1.03, 2.06]*

*Statistically significant at p ≤ 0.05 **Reference category Classification power = 60% Hosmer and Lemeshow test: p = 0.283 Nagelkerke R square = 0.82

Mothers who had 4 or more ANC visits during their last pregnancy were about 4 times more likely to have perceived availability of IDS when compared to mothers who had only one ANC visit during their last pregnancy (AOR = 3.80[1.38, 10.50]). Mothers who live in urban areas were about 2 times more likely to have perceived availability of IDS when compared to their rural counterparts (AOR = 1.74[1.17, 2.59]).

Graduation of a mother’s household as a model family was another variable that was significantly associated with the perceived availability of IDS. Mothers whose households had graduated as a model family were about 1.5 times more likely to have perceived availability of IDS when compared to non-model families (AOR = 1.46[1.03, 2.06]).

### Factors associated with affordability of institutional delivery services

Eight variables were found to be candidates for multivariable logistic regression and after running multivariable logistic regression, three variables were significantly associated with the affordability of IDS. These variables were wealth quintile of mother’s household, number of ANC visits, and husband’s occupation (Table 5).

**Table 5:** Multivariable logistic regression of factors associated with the affordability of institutional delivery services in Jimma Zone, Southwest Ethiopia, 2018

Variables	Ability to afford the cost of IDS		COR (95% CI)	AOR (95% CI)
	No	Yes		
Wealth quintile				
Lowest **	95 (16.0%)	23 (3.9%)	1.0	1.0
Second	64 (10.8%)	55 (9.3%)	3.55 [1.98, 6.34]*	2.83 [1.55, 5.16]*
Middle	64 (10.8%)	55 (9.3%)	3.55 [1.98, 6.34]*	3.04 [1.66, 5.58]*
Fourth	63 (10.6%)	56 (9.4%)	3.67 [2.05, 6.56]*	3.11 [1.71, 5.68]*
Highest	28 (4.7%)	90 (15.2%)	13.27 [7.12, 24.73]*	11.60 [6.02, 22.35]*
Number of ANC visits				
1**	21 (3.6%)	6 (1.0%)	1.0	1.0
2–3	123 (21.0%)	115 (19.7%)	3.27 [1.27, 8.39]*	3.37 [1.31, 8.25]*
≥ 4	162 (27.7%)	159 (27.0%)	3.41 [1.34, 8.68]*	3.48 [1.36, 9.61]*
Husband’s occupation				
Farmer **	208 (35.1%)	205 (34.6%)	1.0	1.0
Merchant	51 (8.6%)	27 (4.6%)	0.53 [0.32, 1.89]	0.44 [0.25, 1.77]
Laborer	41 (6.9%)	10 (1.7%)	0.24 [0.12, 1.50]	0.35 [0.17, 2.76]
Employee	9 (1.5%)	35 (5.9%)	3.94 [1.85, 8.41]*	3.63 [1.51, 8.74]*
Other^**+++**^	5 (0.8%)	2 (0.3%)	0.40 [0.07, 2.11]	0.54 [0.08, 3.32]

*Statistically significant at p ≤ 0.05 **Reference category Classification power = 67.4%

*Hosmer and Lemeshow test: p = 0.50 Nagelkerke R square = 0.74* ^***+++***^*Student, Sheik & Priest*

Mothers in the highest wealth quintile were about 12 times more likely to be able to afford institutional delivery services when compared to mothers in the lowest wealth quintile (AOR = 11.60[6.02, 22.35]). Similarly, mothers in the fourth, middle, and second wealth quintiles each were approximately 3 times more likely to be able to afford institutional delivery services when compared to mothers in the lowest wealth quintile (AOR = 3.11[1.71,5.68], 3.04[1.66, 5.58], and 2.83[1.55, 5.16]), respectively.

Mothers who had four or more ANC visits during their last pregnancy were about 3.5 times more likely to afford institutional delivery services when compared to mothers who had only one ANC visit (AOR = 3.48[1.36, 9.61]). Again, mothers who had 2–3 ANC visits were 3 times more likely to afford institutional delivery services when compared to mothers who had only one ANC visit (AOR = 3.37[1.31, 8.25]).

The husband’s occupation was another variable that was independently associated with the affordability of institutional delivery services. Mothers whose husbands were employed were about 4 times more likely to afford institutional delivery services when compared to mothers whose husbands were farmers (AOR = 3.63[1.51, 8.74]).

### Factors associated with acceptability of institutional delivery services

Seven candidate variables were selected for multivariable logistic regression, and after running multivariable logistic regression, three variables such as mother’s educational level, residence, and graduation of mother’s household as a model family were found to be independently associated with acceptability of IDS (Table 6).

**Table 6:** Multivariable logistic regression of factors associated with acceptability of IDS in Jimma Zone, Southwest Ethiopia, 2018

Variables	Acceptability of IDS		COR (95% CI)	AOR (95% CI)
	No	Yes		
Mother’s educational level				
Can’t read & write**	56 (9.4%)	34 (5.7%)	1.0	1.0
Can read & write	136 (22.9%)	86 (14.5%)	1.04 [0.62, 1.72]	1.06 [0.62, 1.79]
Primary education	99 (16.7%)	65 (11.0%)	1.08 [0.63, 1.83]	1.06 [0.61, 1.84]
Secondary or above	29 (4.9%)	88 (14.8%)	4.99 [2.74, 9.08]*	2.69 [1.42, 5.09]*
Residence				
Rural**	273 (46.0%)	181 (30.5%)	1.0	1.0
Urban	47 (7.9%)	92 (15.5%)	2.95 [1.98, 4.39]*	2.60 [1.66, 4.08]*
HH model status				
No**	213 (35.9%)	100 (16.9%)	1.0	1.0
Yes	107 (18.0%)	173 (29.2%)	3.44 [2.45, 4.83]*	3.12 [2.16, 4.50]*

*statistically significant at p ≤ 0.05 **Reference category Classification power = 67.5% Hosmer and Lemeshow test: p = 0.210 Nagelkerke R square = 0.87

Mothers who have achieved secondary education or above were about 3 times more likely to accept institutional delivery services when compared to mothers who cannot read and write (AOR = 2.69[1.42, 5.09]). Similarly, mothers who live in urban areas were about 3 times more likely to accept institutional delivery services when compared to mothers who live in rural areas (AOR = 2.60[1.66, 4.08]).

Graduation of mother’s household as a model family was also significantly associated with the acceptability of IDS. Mothers whose households were graduated as a model family were 3 times more likely to accept IDS when compared to mothers of non-model families (AOR = 3.12[2.16, 4.50]).

### Qualitative study results

A total of four FGDs were carried out: two with women health development army leaders, one with home-delivered mothers and one with health facility-delivered mothers. Overall, 35 mothers participated in the discussions, and each FGD consisted of 8–10 mothers.

The participants identified many factors that hinder mothers from accessing IDS. The majority of the home-delivered participants reported that there are few health centers in their area, and even the available ones are very far from their homes. They also complained that even if they attend full ANC visits at the nearby health posts in their Kebele, they delivered at home because of the long distances from the health facilities and lack of ambulance services. Participants also reported that home delivery is very common when labor comes at night.

Some of the health facility-delivered mothers raised shortages and/or unavailability of health professionals to give immediate care on arrival. Shortage of prescribed medicines and supplies as well as unavailability of laboratory services were also reported by the majority of the health facility-delivered mothers as problems that affect their intention for subsequent utilization and recommendation for other mothers.

Almost all of the participants in all of the four groups reported difficulty of paying for transport costs and the cost of medicines that are not available in the health centers and bought from private pharmacies. Some mothers also mentioned that their husbands were not voluntary to accompany them to the health centers, while others reported unavailability of somebody else who took care of their house and children when they went to a health center for delivery.

Lack of respect for mothers’ privacy and dignity is the major issue raised by the majority of the health facility-delivered mothers. These are reported as delaying care after arrival, lack of privacy during labor, insulting mothers in labor, hitting mothers in labor, and denying relatives to enter.

## Discussion

The findings of this study showed that moderate proportion of mothers had geographic access to IDS. This finding was similar to findings of other studies conducted in rural Zambia and East Wollega zone in Ethiopia [17, 18].

However, the finding of this study was higher than finding of a study conducted in Tsegedie district of Tigray Regional State of Ethiopia [19]. This difference may be due to the fact that the latter study was conducted 5 years earlier after which many health facilities might have been built close to the communities where mothers lived.

This study found that employed mothers were more likely to have geographic accessibility to IDS when compared to mothers who were farmers. This could be due to the fact that employed mothers live in urban areas where health facilities can be physically accessed within shorter distances. This is also supported by the qualitative finding in which a 28-year old uneducated mother from a rural area who delivered at home said,

*“I am a farmer; I live in a very far village where our farm is located. If I am an educated and government employee, then I would have been living in an urban area where I can easily go to a health facility for delivery.”*

The majority of home-delivered mothers also shared the same point.

Mothers who had 4 or more ANC visits during their last pregnancy were about 4 times more likely to have geographic accessibility to IDS when compared to mothers who had only a single ANC visit during their last pregnancy. This may be due to the fact that mothers who live closer to health facilities attend more ANC visits than mothers who live in remote areas.

A 32-year-old mother who delivered at a health center said,

*“My home is only 10 minutes from the health center on foot walk. I went to the health center for ANC follow-up 5 times before delivery. Even if my labor came at night, I delivered my baby at the health center.”*

But, few home-delivered mothers agreed with the statement of a 40-year old mother,

*“We attend full ANC visits at nearby health posts; but since the health center is very far, we deliver at home especially when labor comes at night.”*

This study also revealed that the number of ANC visits during the last pregnancy was significantly associated with the perceived availability of IDS. This was supported by the qualitative finding in which a 39-year-old mother who delivered at health center said,

*“The nurse who examined me during ANC visit told me many things about the importance of delivering at a health center. Then, I and my husband decided that I have to deliver at the health center. Two weeks before my delivery, I went to the health center to stay at the maternity waiting home. Then, I delivered my baby safely with the help of the nurse.”*

Mothers whose households had graduated as model families were about 1.5 times more likely to have perceived availability of IDS when compared to non-model families. This is evidenced by the qualitative finding in which majority of the discussants in the health development army leaders group said,

*“We are model mothers in our village; we and our families have taken much training from the HEWs and now we are graduated. We have sufficient information about the presence of institutional delivery services at a nearby health center. We also teach our neighbors. If we do not have much information, we should not be model mothers.”*

Mothers in higher wealth quintiles were more likely to be able to afford IDS when compared to mothers in the lowest wealth quintile. This may be due to the fact that households in the higher wealth quintiles have better material and financial assets that enable them to pay/afford IDS without difficulty.

Mothers who had four or more ANC visits during their last pregnancy were also more likely to afford IDS when compared to mothers who had only one ANC visit. This may be due to the fact that mothers who fully attend ANC visits are economically better than those who attend less number of ANC visits. The qualitative finding supported this result, in which a 26-year-old mother who completed primary education said,

*“I have no problem with paying for any cost related to ANC visits or health facility delivery. My husband is a teacher and he earns a monthly salary. We can pay for transport or any related cost”*

Mothers who had attended secondary education and above were more likely to accept IDS when compared to mothers who cannot read and write. This is evidenced by the qualitative finding in which a 43-year-old home-delivered uneducated mother said,

*“My mother and grandmother never knew health facilities. They all had delivered at home with no problems. God was with them. Now I have 6 children and I delivered them all at home without any problem. God, who saved my mother and grandmother, is always with me to help.”*

Similarly, mothers who live in urban areas were more likely to accept institutional delivery services when compared to mothers who live in rural areas. This may be due to the fact that urban mothers are less affected by the negative consequences of cultural beliefs and norms that hinder mothers from accepting institutional delivery as normal and safe. The other possible explanation may be the effect of exposure to different media in urban areas.

Mothers whose households were graduated as a model family were more likely to accept institutional delivery services when compared to mothers of non-model families. The possible explanation for this fact may be the fact that model mothers had gained sufficient information during the training on the 16 packages of the Ethiopian health extension program and their cultural perception towards institutional delivery has positively changed.

This fact was supported by the qualitative finding in which the majority of the discussants in the health-development army leaders group shared the same idea by saying:

*“In the past, we trust Traditional Birth Attendants (TBAs), and we did not go to the health center for delivery. Rather, we delivered at home with the help of TBAs and relatives. However, now we have trained by Health Extension Workers (HEWs), and we are model mothers in the village. We accept and support health facility delivery. We are modern mothers. If not, we are still backward.”*

## Limitations

Since mothers were asked to tell their memories of past experience, there was chance of recall bias and this might have affected the validity of the results. To minimize this bias, only mothers who had given birth recently, (i.e. in the last 6 months preceding the study) were included in the study.

Sampling frame was obtained from health posts registry of delivered mothers. But, since all home delivered mothers may not be registered at the health posts due to different reasons, this may have been resulted in under representation of home delivered mothers in the sample.

The other possible limitation of this study was social desirability bias; because some of the outcome variables were measured by Likert scale. However, it was minimized by the use of negatively worded statements which were reverse coded later during data entry.

Some important variables like previous history of institutional delivery service utilization and participation in support groups like pregnant mothers forums/conferences and health development army meetings were missing in this study. As a result, this study failed to report the effect of these key variables on access to institutional delivery services.

## Conclusions

Moderate proportions of mothers have geographic accessibility to institutional delivery services, but access to institutional delivery services in the other three dimensions of access was low. ANC visits of 4 or above, occupation of a mother when employed, and urban residence were independently associated factors with geographic accessibility to institutional delivery services. ANC visits of 4 or above, urban residence, and graduation of mother’s household as a model family were independently associated with perceived availability of institutional delivery services.

ANC visits of 4 and above, husband’s occupation when employed, and higher wealth quintiles of mother’s household were independently associated with the affordability of institutional delivery services. Urban residence, graduation of mother’s household as a model family, and maternal education of secondary school or above were independently associated with the acceptability of institutional delivery services.

Thus, it was recommended that HEWs or community health workers should strengthen the provision of ANC service to all pregnant mothers and ensure that all mothers who started ANC follow up complete at least 4 visits before their delivery. HEWs should also accelerate the provision of training on the sixteen packages of the Ethiopian Health Extension Program to families and expand the graduation of model households who completes the traing. The District and Zonal Education Offices should give more emphasis to female education to empower women through adult education for short-term solutions and girls education for sustained long-term solutions. The Federal Ministry of Health and Regional Health Bureau should ensure geographic accessibility to all households in the community by building more healthcare facilities as close as possible to the communities. Future researchers should employ pure qualitative study in order to explore the full picture of the factors affecting the four dimensions of access to institutional delivery services. Future researchers are also strongly recommended to include important variables like previous history of institutional delivery service utilization, and participation in support groups like pregnant mothers forums/conferences and health development army meetings which were missing in this study.

## Data Availability

The datasets used and/or analyzed during the current study are available from the corresponding author and can be released upon reasonable request.

## Abbreviations

ANC: Antenatal Care
AOR: Adjusted Odds Ratio
CI: Confidence Interval
COR: Crude Odds Ratio
EDHS: Ethiopian Demographic Health Survey
FGD: Focused Group Discussion
HEW: Health Extension Worker
HH: Household
HSTP: Health Sector Transformation Plan
IDS: Institutional Delivery Service
IRB: Institutional Review Board
MMR: Maternal Mortality Ratio
PCA: Principal Component Analysis
PSU: Primary Sampling Unit
SPSS: Statistical Package for Social Science
SSU: Secondary Sampling Unit
TBA: Traditional Birth Attendant
TSU: Tertiary Sampling Unit
TV: Television

## References

[1] McIntyre D, Thiede M, Birch S (2009). Access as a policy-relevant concept in low- and middle-income countries. Health Econ Policy Law.

[2] Borghi J, Storeng KT, Filippi V (2008). Overview of the costs of obstetric care and the economic and social consequences for households. Stud HSO&P.

[3] Jacobs B, Ir P, Bigdeli M, Annear PL, Van Damme W. Addressing access barriers to health services: an analytical framework for selecting appropriate interventions in low-income Asian countries. Health Policy and Planning. 2012;27(4):288-300.10.1093/heapol/czr038. 10.1093/heapol/czr038 21565939

[4] Kyei-nimakoh M, Carolan-olah M, Mccann TV (2017). Access barriers to obstetric care at health facilities in sub-Saharan Africa — a systematic review. BMC Syst Rev.

[5] Ganle JK, Fitzpatrick R, Otupiri E, Parker M (2016). Addressing health system barriers to access to and use of skilled delivery services : perspectives from Ghana. Int J Health Plann Manage.

[6] Amano A, Gebeyehu A, Birhanu Z (2012). Institutional delivery service utilization in Munisa Woreda , south East Ethiopia : a community based cross-sectional study. BMC Pregnancy Childbirth.

[7] WHO (2014). Maternal mortality fact sheet.

[8] Central Statistical Agency (CSA) [Ethiopia] and ICF (2016). Ethiopia demographic and health survey 2016.

[9] Abeje G, Azage M, Setegn T (2014). Factors associated with institutional delivery service utilization among mothers in Bahir Dar City administration , Amhara region : a community based cross sectional study. Reprod Health.

[10] Ayele DZ, Belayihun B, Teji K, Ayana DA (2014). Factors affecting utilization of maternal health Care Services in Kombolcha District , eastern Hararghe zone , Oromia regional state , eastern Ethiopia. Int Sch Res Not.

[11] Markos D, Odo DB, Shifti DM (2014). Institutional delivery service utilization and associated factors among child bearing age women in Goba Woreda , Ethiopia institutional delivery service utilization and associated factors among child bearing age women in Goba Woreda , Ethiopia. J Gynecol Obstet.

[12] Abera M, Belachew T (2011). Predictors of safe delivery utilization in Arsi zone, south-East Ethiopia. Ethiop J Health Sci.

[13] Fikre AA, Demissie M (2012). Prevalence of institutional delivery and associated factors in Dodota Woreda (district), Oromia regional state. Ethiopia Reprod Health.

[14] Federal Ministry of Health. Health sector transformation plan. Addis Ababa; 2015. Available from: https://www.globalfinancingfacility.org/ethiopia-health-sector-transformation-plan-201516-201920.

[15] WHO (2005). Estimates of unit costs for patient Services for Ethiopia [internet].

[16] WHO (2008). Manual for the household survey to measure access and use of medicines.

[17] Gabrysch S, Cousens S, Cox J, Campbell OMR (2011). The influence of distance and level of care on delivery place in rural Zambia : a study of linked National Data in a geographic information system. PLoS Med.

[18] Feyissa TR, Genemo GA (2014). Determinants of institutional delivery among childbearing age women in Western Ethiopia: unmatched case control study. PLoS One.

[19] Hailu D, Berhe H (2014). Determinants of institutional childbirth service utilisation among women of childbearing age in urban and rural areas of Tsegedie district , Ethiopia. Aust J Midwifery.

